# Disaster Risk Perception and Sustainable Earthquake Awareness Among Public and Private University Nursing Students

**DOI:** 10.1111/phn.13430

**Published:** 2024-09-27

**Authors:** Nurcan Kolaç, Nermin Eroğlu, Cansu Nirgiz

**Affiliations:** ^1^ Department of Public Health Nursing Faculty of Health Sciences, Marmara University Istanbul Türkiye; ^2^ Department of Nursing Faculty of Health Sciences Fenerbahçe University Istanbul Türkiye

**Keywords:** awareness, earthquakes, nursing, perception, risk, students

## Abstract

**Background:**

This research was conducted in descriptive type to determine the disaster risk perception and sustainable earthquake awareness of nursing students studying at public and private universities.

**Methods:**

The study sample consisted of 400 nursing students studying at one public and one private university. The research was conducted between April and May 2023. The data were collected using a Sociodemographic Form, University Students Disaster Risk Perception Scale (USDRPS), and Sustainable Earthquake Awareness Scale (SEAS). Data were collected online using a questionnaire created on Google Forms. Analyses included frequency, percentage, mean, standard deviation values, independent groups *t*‐test, post‐hoc Tukey test, LSD, and ANOVA test.

**Results:**

Of the students, 61.8% had a disaster experience, 17.4% had lost a relative in the disaster, and 76.8% did not consider themselves prepared for a possible disaster. In the study, the exposure sub‐dimension score of the disaster risk perception scale was found to be higher in students who had disaster experience than in those who did not (*p* = 0.032). Nursing students from the private university had higher sustainable earthquake awareness scores than those studying at the state university (*p* = 0.001). The mean scores of female students on the earthquake preparedness and preparation application sub‐dimensions showed a significant difference compared to the scores of male students (*p* = 0.016). In the study, sustainable earthquake awareness total and earthquake preparedness sub‐dimension scores were higher in second‐year nursing students than in students of other years (*p* = 0.042; 0.015). Those who had received disaster training had low scores on the uncontrollable sub‐dimension of the disaster risk perception scale, and high scores on the total SEAS and earthquake‐structure relationship, earthquake preparedness, and earthquake preparation application sub‐dimensions (*p* < 0.05).

**Conclusions:**

In the study, three out of four students did not find themselves prepared for disasters. Those who had disaster experience had higher disaster risk perceptions. Students who had received disaster‐related training had more positive earthquake preparedness, earthquake‐structure relationship, and earthquake preparation applications than those who had not. Students who were female and were in the second year had higher earthquake awareness. Studies can be carried out to inform people about the pre‐disaster and preparation stages to raise awareness about disasters at universities. Education on disaster management can be integrated into courses from the first years of university.

## 1 Introduction

Disasters are events caused by man‐made, technological, or natural hazards that develop suddenly, have devastating effects, result in physical and socioeconomic losses, and occur frequently globally (Cui et al. 2021).

Disasters can affect individuals in particular and societies in general in various ways, depending on how they occur, their effects, and the results they produce. Every year, approximately 68 thousand people die and 218 million are affected due to disasters around the world. The damages caused by disasters, especially by earthquakes, show that society needs to be more aware of disasters (Ünal et al. 2022) because a society that does not know disaster risks and does not have earthquake awareness cannot take measures against them. Societies with disaster risk perception and earthquake awareness can take more measures to prepare for disasters. Risk perception directs individuals to prepare for disasters and to be more alert. Although low‐risk perception may lead to inadequate coping mechanisms, high‐risk perception has been associated with a drive to adopt protective behaviors that are beneficial in supporting greater resilience (Alqahtany and Abubakar 2020; Espina and Teng‐Calleja 2015; Gökçay et al. 2024). Educational institutions are the first to be affected by disasters. They should face disasters, and especially universities should prepare their students for disasters mentally as well as physically. There are studies in the literature conducted to determine the knowledge, attitude, disaster response, preparedness, and self‐efficacy levels of nurses and nursing students toward disasters (Aurelio et al. 2022; Kamanyire et al. 2021; Mohamed, Abdel‐Aziz, and Elsehrawy 2023; Park and Chou 2022; Sattar, Zahra, and Mohamed 2018; Uysal Toraman and Konal Korkmaz 2023). This study was conducted to determine the disaster risk perception and awareness levels of nursing students at public and private universities. Nurses are professionals who come into contact with individuals in healthcare institutions the most, are the first to take part in disasters, and are the most numerous compared to other healthcare professionals (Segev et al. 2024). They undertake important duties in preparing society for disasters and reducing their consequences. For this reason, it has been reported that a 50%–70% reduction in injuries and deaths is achieved thanks to the care and interventions provided by nurses before and after disasters (Firouzkouhi et al. 2021). In this context, nursing students, who will be health professionals of the future, are expected to have a high level of knowledge of disaster risk perception and earthquake awareness, to be prepared for disasters, to find ways to intervene in them, to play critical roles in their management, and to educate and defend society (Bülbül 2021; HUÇEP 2014).

## 2 Methods

### 2.1 The Type of Research

A descriptive design was employed.

### 2.2 Research Questions

- What is the earthquake awareness level of nursing students studying at public and private universities?
- What is the disaster risk perception level of nursing students studying at public and private universities?
- Is there a difference between the socio‐demographic characteristics of nursing students studying at public and private universities and their sustainable earthquake awareness and disaster risk perceptions?

### 2.3 Research Variables

#### 2.3.1 Independent Variables

Socio‐demographic variables such as the university where the students study, age, school year, and gender.

#### 2.3.2 Dependent Variables

Disaster risk perception and earthquake awareness.

### 2.4 The Setting of the Research

The research was conducted between April and May 2023 with students studying in the Nursing Department of the Faculty of Health Sciences of a state and a private university. There were some differences between the curricula of the universities where the research was conducted. Students at the private university took a course called “disaster management and first aid” in the second year. Students at the state university took a course, namely “disaster nursing” as an elective course in the third year.

### 2.5 Population and Sample of the Research

The population of the research consisted of 1314 students. The sample size was calculated as *n *= (1.96)2(0.5)(0.5)/(0.05)2 = 384 using the sampling of the known population method. The research was completed with 400 volunteer students.

### 2.6 Research Inclusion and Exclusion Criteria

The inclusion criteria were volunteering to participate in the study and studying in the nursing department of the universities where the research was conducted. The exclusion criterion was filling out the questionnaire incompletely.

### 2.7 Data Collection Method and Tools

Data were collected online via Google Forms voluntarily. It took approximately 10 min to fill out the questionnaire. 

### 2.8 Socio‐Demographic Form

This form consisted of 17 questions about individuals' age, gender, university, school year, number of people in the house they lived in, disaster experience, disaster training, reading the disaster plan, and participation in disaster drills. The questions were created by the researchers in line with the literature (Avcı 2022; Bülbül 2021; Ünal et al. 2022).

### 2.9 University Students Disaster Risk Perception Scale (USDRPS)

Mızrak and Aslan (2020) developed and did the validity and reliability study of this scale. It is used to measure the disaster risk perception in university students. The score obtained from the 19‐item, five‐point Likert‐type scale with four sub‐dimensions varies between 1 and 5 for each sub‐dimension. The sub‐dimensions are exposure (items 1–6), anxiety (items 7, 8, 9, 14, and 19), effect (items 10, 11, 12, 13, and 15), and uncontrollable (items 16–18). There are no reverse items on the scale. As the score obtained from the scale increases, the perceived risk increases, as well. In this study, Cronbach's alpha value of the scale was found to be 0.94.

### 2.10 Sustainable Earthquake Awareness Scale (SEAS)

This scale was developed by Genç and Sözen (2021) to measure earthquake awareness. It consists of 22 items. As a result of the reliability analysis conducted on the scale, the internal consistency coefficient (Cronbach's alpha) was found as 0.88. Items 20, 21, and 22 have reverse statements. The scale has a five‐point Likert structure and three sub‐dimensions: earthquake structure relationship (4 items), earthquake preparation application (11 items), and earthquake preparedness (7 items). The lowest score is 22 and the highest score is 110. High scores point to increased earthquake awareness. Cronbach's alpha value of the scale was found to be 0.91 in the present study.

### 2.11 Ethical Aspects of the Research

Written permission was obtained for the scales used in the research via e‐mail. Institutional permission was obtained from the nursing departments of the universities where the research data would be collected. Data were collected voluntarily. Written consent was obtained from the participants before data collection was initiated. Ethics committee approval was obtained (30.03.2023/44).

### 2.12 Data Analysis

The data were analyzed on the SPSS 22.0 statistical software by using numbers, percentages, mean and standard deviation values, independent groups *t*‐test, post‐hoc Tukey, LSD, and ANOVA test.

### 2.13 Limitations of the Research

The results of this study are limited to the nursing students who were selected for this study and participated voluntarily. It cannot be generalized to all nursing students.

## 3 Results

As seen in the table, 74% of the students were female, 57.8% were private university students, 29% were in their fourth year, and 43.8% had 3–4 people living in their homes. Of the students, 61.8% had a disaster experience, 17.4% had lost a relative in the disaster, 63.5% had received disaster training, 81.5% wanted to receive disaster training, 58.2% stated that their university had a disaster plan, and 68.7% had not read this disaster plan. It was determined that 62.5% of the students stated they or their family did not have a disaster plan, 83% had participated in a disaster drill before, 76.8% did not find themselves prepared for a possible disaster, 61% did not find the building they lived in safe against disasters, and that 87.2% did not find the people around them aware of disasters (Table 1).

**TABLE 1:** Distribution of the students according to their descriptive characteristics (*n* = 400).

Descriptive characteristics	(*n*)	(%)
**Gender**		
Male	104	26.0
Female	296	74.0
**University**		
Private	231	57.8
Public	169	42.2
**School year**		
1	106	26.5
2	93	23.2
3	85	21.2
4	116	29.0
**Number of households**		
1–2	52	13.0
3–4	175	43.8
5–6	129	32.2
≥7	44	11.0
**Status of having a disaster experience**		
Yes	247	61.8
No	153	38.2
**Status of losing a relative in the disaster (*n* = 247)**		
Yes	43	17.4
No	204	82.6
**Status of having received disaster training**		
Yes	254	63.5
No	146	36.5
**Desire to receive disaster training (*n*** **=** **146)**		
Yes	119	81.5
No	27	18.5
**Whether there is a disaster plan belonging to the university**		
Yes	233	58.2
No	167	41.8
**Status of reading the disaster plan of the university (*n*** **=** **233)**		
Yes	73	31.3
No	160	68.7
**Status of having a disaster plan or the family owning one**		
Yes	150	37.5
No	250	62.5
**Previous participation in disaster drills**		
Yes	332	83.0
No	68	17.0
**Status of feeling prepared for possible disasters**		
Yes	93	23.2
No	307	76.8
**Thinking the building they live in is safe against disasters**		
Yes	156	39.0
No	244	61.0

The regression analysis performed to determine the cause‐effect relationship between sustainable earthquake awareness and disaster risk perception was found to be significant (*F* = 56.462; *p* = 0.000 < 0.05). Sustainable earthquake awareness explained the total variance in nursing students’ disaster risk perception level by 12.2% (*R* ^2 ^= 0.122). It reduced nursing students’ disaster risk perception level (*ß* = −0.352) (Table 2).

**TABLE 2:** The effect of sustainable earthquake awareness on disaster risk perception.

	Unstandardized coefficients		Standardized coefficients	*t*	*p*	%95 Confidence interval	
Independent variable	*B*	SE	*ß*			Lower	Upper
Constant	4.212	0.145		29.111	0.000	3.927	4.496
Sustainable earthquake awareness total	−0.019	0.002	−0.352	−7.514	0.000	−0.024	−0.014

*Notes*: Dependent variable = disaster risk perception, *R *= 0.352; *R^2^ *= 0.122; *F *= 56.462*; p *= 0.000; *Durbin‐Watson value = *1.588.

*B*: unstandardized coefficients; SE: standard error; *ß*: standardized coefficient (SC); *t*: independent groups *t*‐test; *p* < 0.05.

There was no difference between students' gender and disaster risk perception scores (*p* > 0.05). Exposure scores of those studying at the private university were lower (*p* = 0.001 < 0.05). Individuals living with seven or more people in the same hose had the highest total disaster risk perception and exposure, anxiety, and uncontrollable sub‐dimension scores (*p* < 0.05). Effect scores of students living with 1–2 people were the highest (*p* = 0.012). Students who had no disaster experience had a high disaster risk perception exposure sub‐dimension score (*p* < 0.032). The disaster risk perception uncontrollable sub‐dimension score of students who had not received disaster education was high (*p* < 0.007). Those whose university had a disaster plan and those who had a disaster plan or their family had one had low scores on the disaster risk perception total and all its sub‐dimensions (*p* < 0.05). Disaster risk perception anxiety scores of those who had read the disaster plan of their university were high (*p *= 0.02). Students who had participated in disaster drills before, considered themselves prepared for possible disasters, or found the building they lived in safe against disasters had low scores on the disaster risk perception total and all its sub‐dimensions (*p* < 0.05). It was observed that the disaster risk perception total and exposure, effect, and uncontrollable sub‐dimension scores of those who found the people around them conscious of disasters were low (*p* < 0.05) (Table 3).

**TABLE 3:** Variances in students' disaster risk perception scores according to their descriptive characteristics.

Demographic characteristics	*n*	Total disaster risk perception	Exposure	Anxiety	Effect	Uncontrollable
**Number of households**		**Mean ± SD**	**Mean ± SD**	**Mean ± SD**	**Mean ± SD**	**Mean ± SD**
1–2	52	3.360 ± 0.648	3.593 ± 0.761	2.815 ± 0.820	3.696 ± 0.676	3.244 ± 0.938
3–4	175	3.035 ± 0.696	3.355 ± 0.739	2.469 ± 0.863	3.318 ± 0.881	2.869 ± 0.843
5–6	129	3.149 ± 0.773	3.354 ± 0.746	2.592 ± 0.997	3.495 ± 0.943	3.090 ± 0.898
≥7	44	3.401 ± 0.605	3.701 ± 0.595	2.841 ± 0.904	3.650 ± 0.698	3.318 ± 0.670
*F*		4.912	3.951	3.231	3.686	4.999
*p*		0.002	0.009	0.022	0.012	0.002
**Having a disaster experience**						
Yes	247	3.197 ± 0.746	3.486 ± 0.762	2.611 ± 0.955	3.499 ± 0.905	3.090 ± 0.890
No	153	3.086 ± 0.666	3.324 ± 0.688	2.569 ± 0.849	3.399 ± 0.806	2.954 ± 0.834
*t*		1.494	2.148	0.445	1.120	1.523
*p*		0.136	0.032	0.657	0.263	0.129
**Whether the university has a disaster plan**						
Yes	233	3.017 ± 0.656	3.309 ± 0.707	2.433±0.815	3.357 ± 0.857	2.843 ± 0.837
No	167	3.345 ± 0.757	3.584 ± 0.753	2.820 ± 0.999	3.605 ± 0.868	3.311 ± 0.845
*t*		−4.624	−3.732	−4.268	−2.836	−5.503
*p*		0.000	0.000	0.000	0.005	0.000
**Status of having a disaster plan or the family owning one**						
Yes	150	2.972 ± 0.690	3.186 ± 0.749	2.481 ± 0.808	3.309 ± 0.888	2.802 ± 0.847
No	250	3.264 ± 0.712	3.567 ± 0.694	2.662 ± 0.969	3.551 ± 0.846	3.180 ± 0.855
*t*		−4.005	−5.159	−1.922	−2.716	−4.293
*p*		0.000	0.000	0.045	0.007	0.000
**Previous participation in disaster drills**						
Yes	332	3.085 ± 0.671	3.379 ± 0.715	2.492 ± 0.838	3.402 ± 0.847	2.956 ± 0.833
No	68	3.494 ± 0.838	3.645 ± 0.812	3.094 ± 1.100	3.744 ± 0.923	3.441 ± 0.940
*t*		−4.380	−2.731	−5.093	−2.983	−4.279
*p*		0.000	0.007	0.000	0.003	0.000
**Status of feeling prepared for possible disasters**						
Yes	93	2.852 ± 0.687	3.068 ± 0.814	2.398 ± 0.783	3.157 ± 0.857	2.667 ± 0.918
No	307	3.246 ± 0.702	3.532 ± 0.679	2.654 ± 0.945	3.552 ± 0.853	3.151 ± 0.824
*t*		−4.769	−5.496	−2.379	−3.913	−4.830
*p*		0.000	0.000	0.018	0.000	0.000
**Thinking the building they live in is safe against disasters**						
Yes	156	2.948 ± 0.658	3.233 ± 0.729	2.395 ± 0.784	3.268 ± 0.818	2.769 ± 0.843
No	244	3.286 ± 0.724	3.546 ± 0.719	2.722 ± 0.970	3.584 ± 0.880	3.210 ± 0.845
*t *		−4.712	−4.222	−3.538	−3.596	−5.096
*p*		0.000	0.000	0.000	0.000	0.000
**Thinking the people around are aware of disasters**						
Yes	51	2.830 ± 0.773	3.049 ± 0.780	2.467 ± 0.880	3.075 ± 0.905	2.588 ± 0.903
No	349	3.202 ± 0.698	3.479 ± 0.716	2.613 ± 0.920	3.517 ± 0.850	3.104 ± 0.847
*t*		−3.509	−3.953	−1.068	−3.442	−4.028
*p*		0.001	0.000	0.286	0.001	0.000

*Note*: F: ANOVA test; *t*: independent groups *t*‐test; post‐hoc: Tukey, LSD, *p* < 0.05.

There was no difference between nursing students' disaster experience and sustainable earthquake awareness scores (*p* > 0.05). Female participants’ earthquake preparation application scores (*p* = 0.016) were higher than the scores of males (*p* = 0.011). Sustainable earthquake awareness total and earthquake preparedness scores of nursing students studying at a private university were high (*p* = 0.034). Sustainable earthquake awareness total and earthquake preparedness scores of second‐year nursing students were high (*p* = 0.042; 0.015). The earthquake‐structure relationship scores of students living with 1–2 people in the same house were high (*p* = 0.047). Students who had received disaster training, whose university had a disaster plan, who considered themselves prepared for possible disasters, who found the building they lived in safe against disasters, and who found the people around them conscious of disasters had high scores on the sustainable earthquake awareness total and all its sub‐dimensions (*p* = 0.000). Students who had a disaster plan or whose family had one had high scores on the sustainable earthquake awareness total and all its sub‐dimensions (*p* < 0.05). It was determined that the sustainable earthquake awareness total, earthquake‐structure relationship, and earthquake preparation application sub‐dimension scores of those who had previously participated in the disaster drills were high (*p* < 0.05) (Table 4).

**TABLE 4:** Variances in students’ sustainable earthquake awareness scores according to their descriptive characteristics (*n* = 400).

Demographic characteristics	*n*	Sustainable earthquake awareness total	Earthquake‐structure relationship	Earthquake preparation application	Earthquake preparedness
**Gender**		**Mean ± SD**	**Mean ± SD**	**Mean ± SD**	**Mean ± SD**
Male	104	55.683 ± 16.878	11.490 ± 3.773	28.664 ± 9.232	15.529 ± 5.818
Female	296	56.990 ± 12.177	11.993 ± 2.861	31.081 ± 7.118	13.916 ± 4.479
*t*		−0.846	−1.413	−2.747	2.912
*p*		0.469	0.217	0.016	0.011
**University**					
Private	231	57.879 ± 13.030	12.056 ± 3.090	30.801 ± 7.483	15.022 ± 4.764
State	169	54.970 ± 14.087	11.598 ± 3.165	29.976 ± 8.177	13.396 ± 4.955
*t*		2.130	1.451	1.047	3.313
*p*		0.034	0.147	0.296	0.001
**School year**					
1	106	56.396 ± 10.280	11.708 ± 2.608	30.123 ± 5.798	14.566 ± 4.573
2	93	59.667 ± 14.411	12.398 ± 3.311	31.742 ± 8.113	15.527 ± 5.094
3	85	56.882 ± 15.468	11.824 ± 3.395	31.012 ± 8.888	14.047 ± 5.094
4	116	54.293 ± 13.646	11.603 ± 3.192	29.310 ± 8.136	13.379 ± 4.745
*F*		2.765	1.268	1.906	3.539
*p*		0.042	0.285	0.128	0.015
**Number of households**					
1–2	52	59.635 ± 11.157	12.808 ± 2.536	32.481 ± 6.569	14.346 ± 4.842
3–4	175	57.520 ± 14.324	11.880 ± 3.308	30.823 ± 8.425	14.817 ± 4.847
5–6	129	54.302 ± 13.597	11.388 ± 3.143	29.155 ± 7.530	13.760 ± 4.953
≥7	44	56.546 ± 12.001	12.068 ± 2.748	30.386 ± 6.672	14.091 ± 5.043
*F *		2.400	2.675	2.533	1.193
*p *		0.067	0.047	0.057	0.312
**Having a disaster experience**					
Yes	254	59.189 ± 13.444	12.484 ± 2.946	31.689 ± 7.471	15.016 ± 5.045
No	146	52.233 ± 12.596	10.781 ± 3.146	28.301 ± 7.874	13.151 ± 4.426
*t *		5.097	5.431	4.280	3.719
*p*		0.000	0.000	0.000	0.000
**Whether the university has a disaster plan**					
Yes	233	60.378 ± 12.442	12.661 ± 2.812	32.794 ± 6.879	14.923 ± 4.920
No	167	51.449 ± 13.341	10.749 ± 3.209	27.186 ± 7.815	13.515 ± 4.781
*t *		6.867	6.321	7.594	2.855
*p*		0.000	0.000	0.000	0.005
**Status of having a disaster plan or the family owning one**					
Yes	150	61.560 ± 12.454	12.660 ± 2.910	33.120 ± 7.158	15.780 ± 5.011
No	250	53.704 ± 13.341	11.384 ± 3.159	28.852 ± 7.719	13.468 ± 4.640
*t *		5.844	4.027	5.500	4.681
*p*		0.000	0.000	0.000	0.000
**Previous participation in disaster drills**					
Yes	332	57.934 ± 12.796	12.145 ± 2.884	31.392 ± 7.286	14.398 ± 4.888
No	68	50.382 ± 15.355	10.485 ± 3.846	25.868 ± 8.539	14.029 ± 5.016
*t*		4.278	4.064	5.525	0.563
*p*		0.000	0.001	0.000	0.574
**Status of feeling prepared for possible disasters**					
Yes	93	65.301 ± 13.577	13.527 ± 3.060	34.290 ± 8.044	17.484 ± 5.204
No	307	54.029 ± 12.418	11.358 ± 2.972	29.290 ± 7.330	13.381 ± 4.395
*t *		7.501	6.123	5.632	7.544
*p *		0.000	0.000	0.000	0.000
**Thinking the building they live in is safe against disasters**					
Yes	156	61.256 ± 13.651	13.058 ± 2.956	32.462 ± 7.699	15.737 ± 5.314
No	244	53.705 ± 12.653	11.098 ± 2.995	29.168 ± 7.579	13.439 ± 4.409
*t *		5.644	6.414	4.213	4.689
*p *		0.000	0.000	0.000	0.000
**Thinking the people around are conscious of disasters**					
Yes	51	67.059 ± 14.412	13.588 ± 3.232	35.569 ± 8.446	17.902 ± 5.209
No	349	55.129 ± 12.742	11.610 ± 3.034	29.705 ± 7.405	13.814 ± 4.643
*t *		6.139	4.313	5.185	5.780
*p *		0.000	0.000	0.000	0.000

*Note*: F: ANOVA test; *t*: independent groups *t*‐test; post‐hoc: Tukey, LSD; *p* < 0.05.

## 4 Discussion

Türkiye is a country where earthquakes occur frequently due to major fault lines. Two major earthquakes occurred in the country on February 6, 2023, at 04.17: a 7.8 magnitude earthquake with the epicenter of Pazarcık district of Kahramanmaraş province, lasting 65 s, and a 7.6 magnitude earthquake with the epicenter of Elbistan district of the same city, lasting 45 s, both of which caused the death of 50,783 people and loss of property in 11 provinces (Gürboğa, Kayadibi, and Akıllı 2024; Zhang et al. 2023). These earthquakes also affected Syria, one of the neighboring countries. In Syria, 8476 people lost their lives. More than 40 thousand aftershocks followed. The Kahramanmaraş earthquake was the longest and most severe in recent history in Türkiye. In the aftermath of the earthquake, basic needs, such as life safety, shelter, nutrition, and access to health services created significant problems (Genç Köse, Gümüşler Başaran, and Kefeli Çol 2024; Segev et al. 2024).

Although more than a year has passed since the earthquake, there are still many problems in the region that affect the return to normal (Demir Yıldız and Demir Öztürk 2023). In this earthquake, a total of 1361 university students, 115 of whom were foreigners, lost their lives. It was determined that 106 of the 18 university buildings in 11 provinces were heavily damaged, 410 were slightly damaged, and 606 were undamaged (Anadolu Agency 2023).

As a result of the losses experienced in all disasters in Türkiye and the nearby geography, the resilience of individuals rather than the protective practices of the state has shown the necessity of taking responsibility for disaster awareness and possible risks (Doğru and Coşkun 2023; Turan et al. 2021). In this study, as nursing students' awareness of earthquakes increased, their disaster risk perception levels decreased. This is a valuable result in terms of increasing resilience and preparedness for disasters in nursing students (Abou Hashish 2023; Xu et al. 2020). Nursing students have disaster awareness, but they think that they may be harmed and cannot protect themselves in the event of a possible disaster, and they do not think the environment they live in is resistant to disasters (Hung et al. 2021; Kang, Lee, and Seo 2022). Studies conducted in Türkiye showed that the knowledge level of nurses and nursing students about disaster nursing practices was inadequate and their self‐efficacy was at a moderate level (Uysal Toraman and Konal Korkmaz 2023). Similarly, a study revealed that 64.3% of nursing students in Iran had not received any training on disasters and that 88.6% of them had never participated in disaster drills and therefore were not prepared for disasters (Kaviani et al. 2022). This shows that nursing students need to participate in practices that provide more knowledge and skills about disasters and disaster nursing and increase their self‐confidence.

In this study, it was seen that nursing students were ready for “earthquake preparation application” the most. Similarly, in a study conducted by Budak and Kandil (2023) with university students, students' awareness of “earthquake preparation application” was found to be high. Another study conducted in America showed that university students were prepared for earthquakes (Longo 2022). Based on this finding, students' awareness of the measures to be taken against a possible earthquake and understanding its effects when it occurred was at a good level. Spatial situations are thought to affect risk perception. The study was conducted in a country/region at risk for earthquakes (Marmara Region); therefore, it is thought that students in particular may have been affected by their emotions, experiences, and the destructive nature of earthquakes.

No significant difference was found between gender and disaster risk perception in this study. However, just as there are studies in the literature showing that disaster risk perception does not vary by gender (Paul and Bhujyan 2010), there are also those indicating that females’ disaster risk perception is higher than that of males. Many studies have identified gender as a notable socio‐demographic determinant of earthquake risk perception. The results of the current study revealed that males had lower risk perception (Cvetković, Adem, and Ivanov 2019; Mallick et al. 2022; Mills et al. 2016; Niforatos, Panagiotakos, and Delladetsimas 2024). A study conducted in America showed that females found environmental risks more threatening than males (Niforatos, Panagiotakos, and Delladetsimas 2024; Song 2014). At the same time, the mean scores of female students on earthquake preparedness and earthquake preparation application sub‐dimensions were found to be higher than those of males in this study. Budak and Kandil (2023) found that males’ earthquake‐structure relationship scores were higher than those of females, while Sözen and Genç (2023) reported just the opposite. Similarly, in a study conducted in Greece, the earthquake perception score was found to be low in males (Niforatos, Panagiotakos, and Delladetsimas 2024). In the literature, risk perception has been found as one of the determinants that affect individuals' behaviors and attitudes (positively or negatively). It has been reported in the literature that females’ earthquake preparedness and risk perceptions are affected by their social and caring roles and that they are especially more concerned about health and safety issues (Niforatos, Panagiotakos, and Delladetsimas 2024).The high number of female students in both universities where the study was conducted and the fact that their universities were located in a region where earthquakes occurred frequently can be explained by the fact of the recent earthquake.

The relationship between having disaster experience and the exposure sub‐dimension of the disaster risk perception was significant. A study conducted in Pakistan showed that past disaster experiences significantly affected risk perception (Rana, Jamshed, and Younas 2020). After a disaster in the Czech Republic, individuals became more aware of possible risks and took them more seriously (Bera and Daněk 2018). According to the results of a study conducted with individuals living in different countries in the European Union, exposure to hazards was found to increase risk perception (Knuth et al. 2014; Niforatos, Panagiotakos, and Delladetsimas 2024). Another study conducted in New Zealand similarly revealed that disaster experience increased risk perception (Lawrence, Dorothee, and Julia 2014). In a study conducted in China, it was stated that people who experienced disasters had a higher earthquake risk perception (Cui, Han, and Wang 2018). Disaster experiences leave permanent and deep traces on individuals. For example, those who successfully escape a disaster may have greater confidence in their abilities and believe that the impact of disasters will be controlled through their own efforts (Yang, Tan, and Peng 2020). It has been stated that personal experiences affect people's risk perceptions (Alqahtany and Abubakar 2020; Cui, Han, and Wang 2018). This may mean that people who experience a disaster are more ready to take measures and protective actions against future threats.

In this research, the disaster risk perception scores of nursing students who had previously participated in disaster drills were found to be low, but their earthquake awareness scores were high. It was observed that students who had participated in disaster drills in educational institutions in the Tohoku, Kansai, and Chubu regions of Japan and Chile were better prepared for disasters (Bhandari, Rahman, and Takahashi 2023; Reyes 2010). Disaster risk perceptions of students who have not participated in disaster drills before may also increase because they do not know what to do in the event of a real disaster. In this study, nursing students who did not find the building they lived in safe against disasters had high levels of disaster risk perception. Studies conducted in Pakistan indicated that the increase in the physical fragility of houses led to an increase in individuals' disaster risk perception (Khan, Qureshi, and Rana 2019; Qureshi et al. 2021). When students do not find the building they live in physically strong enough, this may strengthen their feeling of unpreparedness for disasters.

In this study, one‐third of nursing students did not consider themselves prepared for disasters and stated that they and their families did not have a disaster plan. The disaster risk perception level of students who did not have a disaster plan that belonged to them, their university, and their families and who did not consider themselves prepared for possible disasters increased. In the further analysis performed in this study, disaster risk perception was found to be associated with sustainable earthquake awareness (Table 2). Studies conducted with university students in Türkiye showed that students were not adequately prepared for possible earthquakes (Budak and Kandil 2023; Sözen and Genç 2023; Uysal Toraman and Konal Korkmaz 2023). Students studying at the private university were better at sustainable earthquake awareness and earthquake preparedness. Those studying at the state university had a high level of disaster exposure perception. It is thought that students' disaster awareness may have increased because more disaster‐related activities and drills were held in the private university than in the public university. Another finding was that second‐year students' sustainable earthquake awareness and earthquake preparedness scores were higher. This may have been related to the individual interest and knowledge of second‐year nursing students at the private university where the research was conducted. Studies highlight that training on earthquake preparedness and disaster awareness is important (Sakurai, Sato, and Murayama 2020; Segev et al. 2024; Seong, Ryu, and Sok 2023).

It has been reported in the literature that the increase in the number of people living together in the same house can positively affect safe evacuation in case of a disaster (Tam, Huang, and Chan 2018; Valladares‐Garrido, Zapata‐Castro, and Valdiviezo‐Morales 2022). In this research, as the number of people living in the same house increased, the level of disaster risk perception increased, as well. This is a positive finding in that students who are more concerned about disaster risks are more likely to take measures and follow emergency procedures. In other words, the participants of this study may have been influenced by the fact that individuals living in the same house would help each other during a disaster and possibly help recover from its negative effects, or that they had a responsibility to ensure the safety and meet the needs of more people. However, in a study conducted in Pakistan, disaster risk perception decreased as the number of people living in the same house increased (Rana, Jamshed, and Younas 2020). Also, in a study conducted with older individuals in Iran, it was found that the number of people in the house was not effective (Hattori et al. 2021). For this reason, it is thought that developing a proactive attitude toward the risks of disasters in all segments of society can be effective.

Students who had received disaster‐related training had low scores on the uncontrollable sub‐dimension of the disaster risk perception scale, while earthquake awareness scores were high. This result indicates that students who receive disaster‐related training have high levels of awareness about their capacity to protect themselves when a disaster occurs and implement disaster plans successfully. Similar results were found in the literature (Doğru and Coşkun 2023; Ertuğrul and Ünal 2020). In addition, in the study by Budak and Kandil (2023), disaster awareness levels of those who had attended disaster‐related training were higher. Studies conducted in Japan and Chile indicated that the training given to healthcare professionals had a positive effect on disaster preparedness (Bhandari, Rahman, and Takahashi 2023; Reyes 2010). Similarly, in a study conducted in Greece, it was found that individuals' knowledge and experience about earthquakes had a significantly positive relationship with earthquake risk perception (Niforatos, Panagiotakos, and Delladetsimas 2024). Based on these findings, it is expected that universities train nursing students with the same basic knowledge, attitudes, and skills. According to the report prepared by the International Council of Nurses (ICN), disaster nursing education is a global necessity and it should become a part of nurses' knowledge and skills in disaster preparation, intervention, and recovery stages (ICN, 2024). Recently, the high loss of life in devastating earthquakes in countries such as Iran, Greece, and Türkiye, which are in the same earthquake zone, has increased not only national but also international awareness. It is expected to prepare for disasters and build resilience to disasters to achieve the 2030 Development Goals (Republic of Türkiye, Strategy and Budget Presidency, 2024). Universities can play an important role in focusing students on disaster preparedness and encouraging and strengthening disaster management activities in society. There are very few studies focusing on disaster preparedness in Türkiye (Uysal Toraman and Konal Korkmaz 2023). Disaster preparedness can be carried out effectively through planned response and action programs. For this purpose, informing society and continuing campaigns are positive steps but they may not be enough alone. The education given to undergraduate nursing students at universities is expected to influence and motivate their attitudes. There is strong evidence in the literature on the positive impact of formal university education on disasters in reducing vulnerability (Abou Hashish 2023; Longo 2022; Sakurai, Sato, and Murayama 2020; Seong, Ryu, and Sok 2023). Nursing undergraduate programs in Türkiye are carried out in state and private educational institutions with different structures, including Nursing Departments of Health Schools, Nursing Schools, Nursing Departments in the Faculty of Health Sciences, and Nursing Faculties. There are differences between these nursing education programs in terms of the workload stipulated in national and international legislation, especially in terms of theoretical and applied course hours. In the public school where this research was conducted, the relevant education was given in an elective course in the third year under the name, “disaster nursing.” At the private university, it was given in a course in the second year under the name, “disaster management and first aid.” In addition, the roles and responsibilities of the public health nurses were mentioned in the disasters and disaster management subjects, which were given in the “public health nursing” course in the senior year of both nursing departments. In our country, the Nursing National Core Education Program (HUCEP‐2014) aims to include “planning, implementation, management, and evaluation before, during, and after an emergency” in the content of vocational courses and to impart students’ knowledge and skills. However, there are deficiencies in creating disaster nursing education and training programs in the nursing education curriculum in Türkiye. It has been reported that nursing students in Türkiye receive different education and disaster nursing is not widely included in the curriculum, which may affect the psychomotor skills and self‐efficacy of nursing students regarding disasters (Uysal Toraman and Konal Korkmaz 2023). However, it is thought that students can be more competent at every stage of a disaster if they have both the necessary knowledge and skills.

## 5 Conclusions

In the study, as nursing students' earthquake awareness increased, their risk perceptions decreased. However, three out of four participants did not find themselves prepared for disasters. Despite this, the students had not read the disaster plan of their university and had not created a disaster plan for their family, either. This shows that more emphasis should be placed on the preparedness phase of disaster management. More information on pre‐disaster and preparation stages can be included in action plans aimed at creating a disaster culture in campus environments. If courses on natural disasters are included in universities, students can use this information to understand the natural disasters they may encounter and raise public awareness about them. It is recommended to add relevant courses to the education curricula, to activate student communities on disaster management at universities, and to increase students’ awareness through promotions, seminars, conferences, and exercises about what to do in disasters on campus.

## Data Availability

The data that support the findings of this study are available from the corresponding author upon reasonable request.
